# Editorial: Chronic hepatitis B management: current status and future directions

**DOI:** 10.3389/fmed.2025.1638241

**Published:** 2025-06-24

**Authors:** Ivana Lazarevic, Krzysztof Tomasiewicz

**Affiliations:** ^1^Institute of Microbiology and Immunology, Faculty of Medicine, University of Belgrade, Belgrade, Serbia; ^2^Department of Infectious Diseases and Hepatology, USK-1, Medical University of Lublin, Lublin, Poland

**Keywords:** hepatitis B virus (HBV), chronic hepatitis B (CHB), antiviral therapy, diagnostic methods, vaccination, new therapeutic strategies, new biomarkers

Infection with the hepatitis B virus (HBV) remains a significant global health challenge. According to the latest estimates, approximately two billion people worldwide have been exposed to HBV, and 254 million have a persistent infection that predisposes them to develop severe complications, including liver cirrhosis, hepatic decompensation, and hepatocellular carcinoma (HCC) (1, 2). According to the latest EASL guidelines, based on specific virological, immunological, and clinical features, persistent HBV infection evolves through two non-successive phases of chronic infection and two phases of chronic hepatitis B (CHB) (3). Our understanding of chronic HBV infection has evolved over the years, leading to significant advancements in its diagnosis, management, and prevention. The introduction of efficient vaccination and universal vaccination programs has played a crucial role in preventing new infections and reducing the global burden of infection. The development of effective antiviral therapies has revolutionized the management of CHB by reducing liver inflammation and preventing liver-related complications. Additionally, developing new diagnostic techniques and identifying new biomarkers has facilitated clinical decision-making in managing CHB.

The persisting challenges in CHB management include the inability to achieve a sterilizing cure with current therapies, antiviral drug resistance, long-term safety concerns regarding antiviral treatments, and difficulty reaching vulnerable populations in vaccination programs. This Research Topic was designed to gather experiences with new diagnostic approaches, innovative pre-clinical and clinical therapeutic strategies, various monitoring techniques, and the impact of vaccination programs on disease prevention and control. It encompasses eight original articles, one brief report, two clinical trials, one case report, one systematic review with meta-analysis, and one narrative review.

The primary goal of CHB therapy is to improve long-term outcomes by consistently inhibiting HBV replication. However, current treatments, pegylated interferon α (PEG-IFNα) and nucleos(t)ide analogs (NAs), cannot achieve a sterilizing cure because of their inability to eliminate the HBV mini-chromosome in hepatocyte nuclei or viral DNA integrated into the host genome. Therefore, the alternative goal is a “functional cure,” characterized by persistently undetectable serum HBV DNA and hepatitis B surface antigen (HBsAg), potentially with seroconversion to the corresponding antibodies (3, 4). Other problems with current therapies include their potential adverse effects and the selection of biomarkers capable of predicting a functional cure. The research by Lv et al. confirmed that the combined therapies with NAs and Peg-IFN-α-2b have synergistic and complementary effects, leading more frequently to a clinical (functional) cure. The study also identified two independent predictors of a functional cure - baseline HBsAg level and total bilirubin at 24 weeks. A clinical trial by Zhao W. et al. demonstrated that tenofovir alafenamide (TAF) had minimal effect on the lipid profiles of patients with CHB and that it was safe in subjects with fatty liver disease and hypercholesterolemia. Since the safety and efficacy of PEG-IFN-α for patients with HBV-related cirrhosis has not been extensively researched, a clinical trial by Wang et al. aimed to investigate these factors. Despite a few patients who had to discontinue therapy due to severe adverse effects, the overall conclusion was that this therapy was well-tolerated and reduced HBsAg levels without accelerating disease progression. This is particularly important as new class medications (e.g., bepirovirsen) may require immunomodulators such as PEG-IFN-α for better efficacy. Yakut performed a retrospective study showing that the relative risk of cardiovascular disease was increased in patients using entecavir (ETV).

Considerable heterogeneity characterizes the natural history of HBV infection, and a significant proportion of individuals with chronic HBV infection cannot easily be classified into the four phases proposed by the guidelines. Based on different levels of HBeAg, ALT, and HBV DNA, Zhang et al. categorized untreated patients whose profiles did not fit the conventional phases into four “gray zones” to analyze the probability of HCC development. The risk of HCC was high in all gray zone patients, with the presumable explanation that the majority of gray zone patients have significant underlying histological disease (5). The link between HBV infection and hepatic lipid metabolism has recently attracted considerable attention (6). A study by Fang et al. demonstrated that fatty liver disease, which is often found alongside CHB, does not influence the progression of liver fibrosis, although it may be reflected in increased ALT levels. Overt hepatic encephalopathy (OHE), a complex neurological manifestation in patients with HBV-induced cirrhosis, is associated with systemic inflammation and lipid abnormalities. In light of this, Shi et al. emphasized the prognostic value of inflammatory lipid biomarkers for mortality in OHE patients.

Upon entering the nucleus, HBV DNA forms a key molecule—covalently closed circular DNA (cccDNA), an epigenetically regulated mini-chromosome that is responsible for viral persistence. After discontinuing NA treatment or immunosuppression, viral replication frequently resumes from cccDNA, indicating that it can last indefinitely (7). Quantitation of viral cccDNA in hepatocytes is the gold standard for gaining information on HBV replicative and transcriptional activity (8, 9). However, its routine use as a biomarker is limited by the necessity of a liver biopsy. Xu W. et al. reported on a female patient whose liver biopsy tested positive for HBV cccDNA and pregenomic RNA (pgRNA) 7 years after achieving a functional cure and 5 years after HBsAg seroconversion. Interestingly, all viral biomarkers in the blood were negative, indicating that non-invasive biomarkers reflecting the persistence of cccDNA are unreliable. Zhao S. et al. found that intrahepatic levels of HBV cccDNA in untreated patients, along with HBV-DNA level, HBeAg, and HBsAg, were substantially higher during HBeAg-positive phases. This is due to increased viral protein synthesis and release, which is usually accompanied by hepatocyte damage and histologic changes (10). The study confirmed that elevated intrahepatic HBV cccDNA levels increase the likelihood of hepatic inflammation. Finally, a narrative review by Saeed et al. thoroughly explored current and emerging antiviral therapeutic approaches, particularly those aimed at targeting cccDNA as a key player in viral persistence.

New biomarkers are constantly needed to reflect infection activity and aid in decision-making during CHB. Serum HBV RNA consists mainly of pgRNA and can be a surrogate marker of cccDNA transcriptional activity (4, 8, 9). A study by Hao et al., comprising untreated CHB patients, confirmed that serum RNA was more frequently found in HBeAg-positive phases and varied with HBsAg levels. At the same time, HBV DNA showed no difference based on HBeAg status since it can also originate from integrated HBV DNA. The importance of HBV RNA as a biomarker was further demonstrated by the detection of small amounts of RNA in an HBV DNA-negative patient. Since host biomarkers should complement viral biomarker findings in clinical decision-making, the meta-analysis by Manea et al. presented correlations between cytokines and HBV DNA levels. On an extensive range of 32 cytokines, the study identified IL-9 and IL-10 as reflecting HBV DNA levels.

Children exposed to HBV are more likely to develop CHB, which increases their lifelong risk of adverse outcomes. A retrospective study by Xu Y. et al. demonstrated a considerable prevalence of coexisting HBsAg and anti-HBs antibodies in pediatric CHB patients, highlighting the importance of this specific serological pattern. One possible explanation for this rare phenomenon is HBV genetic variability, which is responsible for HBV escape mutants that the corresponding antibodies cannot neutralize (11). Another possible explanation is the immune system's inability to respond due to immunosuppression or immaturity (12).

The most effective way to prevent HBV infection is active immunization. However, despite the high protection rates in vaccinated individuals, 5–10% of individuals do not respond to the vaccine, and this percentage varies significantly among special population groups. Several new vaccines are designed to protect highly vulnerable individuals (13). Patients undergoing parenteral therapies for chronic kidney disease (CKD) are at an elevated risk of HBV infection and typically exhibit a weaker immune response to vaccination than immunocompetent individuals. Hernán-García et al. aimed to evaluate the response to the Fendrix^®^ vaccine and the antibody persistence in CKD patients. Fendrix^®^ is a yeast-derived recombinant small surface antigen vaccine adjuvanted with the AS04C system, which is a toll-like receptor 4 (TLR4) agonist. The study confirmed the high seroconversion rates reported for adjuvanted vaccines. High initial antibody levels did not ensure sustained levels over time, highlighting the importance of periodic serological monitoring and booster doses for non-protective responses.

These articles provide new insights into the benefits and limitations of current and innovative therapeutic strategies while assessing the prognostic value of traditional and novel viral and host biomarkers. The presented results indicate that CHB remains a significant health issue despite advancements in therapy and diagnostic methods and will continue to be a focus of research in the foreseeable future.
